# Qualitative and Quantitative Assessments of Blood Flow on Tears in Type B Aortic Dissection With Different Morphologies

**DOI:** 10.3389/fbioe.2021.742985

**Published:** 2021-10-08

**Authors:** Simone Saitta, Baolei Guo, Selene Pirola, Claudia Menichini, Daqiao Guo, Yan Shan, Zhihui Dong, Xiao Yun Xu, Weiguo Fu

**Affiliations:** ^1^ Department of Chemical Engineering, Imperial College London, London, United Kingdom; ^2^ Department of Electronics Information and Bioengineering, Politecnico di Milano, Milan, Italy; ^3^ Department of Vascular Surgery, Zhongshan Hospital, Institute of Vascular Surgery, Fudan University, Shanghai, China; ^4^ Department of Radiology, Zhongshan Hospital, Fudan University, Shanghai, China

**Keywords:** type B aortic dissection, 4D flow MRI, aortic remodeling, flow imaging, thoracic endovascular aortic repair (TEVAR)

## Abstract

**Objective:** The interactions between aortic morphology and hemodynamics play a key role in determining type B aortic dissection (TBAD) progression and remodeling. The study aimed to provide qualitative and quantitative hemodynamic assessment in four different TBAD morphologies based on 4D flow MRI analysis.

**Materials and Methods:** Four patients with different TBAD morphologies underwent CT and 4D flow MRI scans. Qualitative blood flow evaluation was performed by visualizing velocity streamlines and flow directionality near the tears. Quantitative analysis included flow rate, velocity and reverse flow index (RFI) measurements. Statistical analysis was performed to evaluate hemodynamic differences between the true lumen (TL) and false lumen (FL) of patients.

**Results:** Qualitative analysis revealed blood flow splitting near the primary entry tears (PETs), often causing the formation of vortices in the FL. All patients exhibited clear hemodynamic differences between TL and FL, with the TL generally showing higher velocities and flow rates, and lower RFIs. Average velocity magnitude measurements were significantly different for Patient 1 (*t* = 5.61, *p* = 0.001), Patient 2 (*t* = 3.09, *p* = 0.02) and Patient 4 (*t* = 2.81, *p* = 0.03). At follow-up, Patient three suffered from left renal ischemia because of FL collapse. This patient presented a complex morphology with two FLs and marked flow differences between TL and FLs. In Patient 4, left renal artery malperfusion was observed at the 32-months follow-up, due to FL thrombosis growing after PET repair.

**Conclusion:** The study demonstrates the clinical feasibility of using 4D flow MRI in the context of TBAD. Detailed patient-specific hemodynamics assessment before treatment may provide useful insights to better understand this pathology in the future.

## Introduction

Approximately 30% of patients with aortic dissection have one or more ischemic complications of the peripheral vasculature, including stroke, paraplegia, loss of peripheral pulses, and compromised renal or mesenteric perfusion (Nienaber et al., 2016). With regards to Stanford type B aortic dissection (TBAD), the presence of a dissecting aneurysm or the occurrence of organ ischemia requires immediate treatment. In recent years, primary entry closure with thoracic endovascular aortic repair (TEVAR) has been performed as the first-line strategy for complicated TBAD (Nienaber et al., 2013). The primary goal of TEVAR is to reduce flow and pressure in the false lumen (FL). Aortic remodeling is facilitated in most cases, causing the FL to regress and thrombose (Nienaber et al., 2013). However, this is a highly complex pathology and each dissection has the anatomical morphology of its own, with different dissection propagation lengths, numbers of secondary tears, volumes of true lumen (TL) and FL and even some cases with multiple FLs. These morphological features are of critical importance for aortic remodeling in both acute and chronic dissections and are strictly related to hemodynamics (Xu et al., 2017).

Limited by the complex interactions between hemodynamics and geometry in TBAD, little effort has been spent on understanding the hemodynamic mechanisms leading to visceral ischemia and asymptomatic cases with malperfusion following the primary entry tear (PET) closure. Moreover, current risk stratification methods rely on anatomic rather than hemodynamic characteristics (Evangelista et al., 2012; Allen et al., 2019). Incorporating hemodynamics in patient risk stratification could improve TBAD development predictions and, eventually, treatment (François et al., 2013).

Until recently, hemodynamic parameters could not be easily measured, and patient-specific flow alterations are not yet clearly understood. Sophisticated numerical simulations studies have evaluated blood flow in TBAD using computational fluid dynamics (CFD) and four-dimensional phase-contrast magnetic resonance imaging (4D flow MRI) (Pirola et al., 2019). In particular, 4D flow MRI allows to measure blood flow velocities in the aorta at any anatomic location of interest (Markl et al., 2016), enabling quantitative functional assessment of blood flow (Youssefi et al., 2017; Soulat et al., 2020). Based on this imaging technique, previous studies have demonstrated the feasibility of evaluating hemodynamic alterations in the TL and FL (François et al., 2013; Liu et al., 2018; Allen et al., 2019). These similar studies provide valuable information about the complex hemodynamics involved in TBAD. Nonetheless, they did not provide insights into how much hemodynamics can vary among patients with different aortic morphologies. In fact, despite the constant research efforts, the complex interactions between patient-specific hemodynamics and aortic morphology make it difficult to fully understand the causalities of this disease and to relate such factors to TBAD progression. It is thus necessary to further investigate patient-specific TL and FL hemodynamic alterations. Accordingly, 4D flow MRI assessment of blood flow in TBADs may improve patient risk assessment and allow additional procedures to be targeted to patients with organ malperfusion (Adriaans et al., 2019; Zilber et al., 2021).

In the present study, four acute TBAD patients with different aortic morphologies were evaluated through 4D flow MRI analysis and aortic remodeling after TEVAR was assessed at 12-, 24- or 32 months follow-up. The main goal of this study was to analyze pre-TEVAR blood flow dynamics in the TL and FL of a diverse set of aortic morphologies, with special attention to flow distributions at the entry/re-entry tear (RT) sites. Additionally, we provide new insights into the possible relationships between pre-TEVAR aortic hemodynamics and aortic remodeling that occurred following TEVAR, suggesting that lower FL flow rates concurrently with branch vessel perfusion through RTs may represent a risk factor for the development of malperfusion after TEVAR.

## Materials and Methods

### Study Design

In this study, a total of four patients (45–59 years-old, 3 males and 1 female) with acute complicated TBAD were included. Preoperative computed tomography (CT) and 4D flow MRI scans were performed on all patients. Different aortic morphologies were observed among the four cases during CT imaging analysis. The geometric parameters before TEVAR are reported in Table 1. This study was approved by the Ethics Committee of Zhongshan Hospital, Fudan University, Shanghai, China (Ethics approval number Y2017-056).

**TABLE 1:** Aortic dissection morphologic measurements of interest for the four patients.

Geometric variable	Patient 1	Patient 2	Patient 3	Patient 4
PET maximum diameter (mm)	22	10	4 (FL1) + 5 (FL2)	11.3
RT maximum diameter (mm)	10.01 (RT1)	4.89 (RT1)	9.31 (RT1; FL1 to TL)	4.24 (RT1) 7.03 (RT2)
		4.66 (RT2)		
		4.91 (RT3)		
Celiac artery	TL	FL	TL	FL
Superior mesenteric artery	TL	FL	TL	TL
Right renal artery	FL	TL	TL	TL
Left renal artery	TL	TL + FL	TL (dynamic ischemia)	FL
Maximum FL cross-sectional area (mm^2^)	684	467	305 (FL1) + 827 (FL2)	970
TL volume (cm^3^)	18	49.1	44.8	86.0
FL volume (cm^3^)	81	143	64.3 (FL1), 159.4 (FL2)	198.7

FL, false lumen; PET, primary entry tear; RT, re-entry tear; TL, true lumen.

### Computed tomography Scanning Protocol and Post-Processing

**FIGURE 1:**
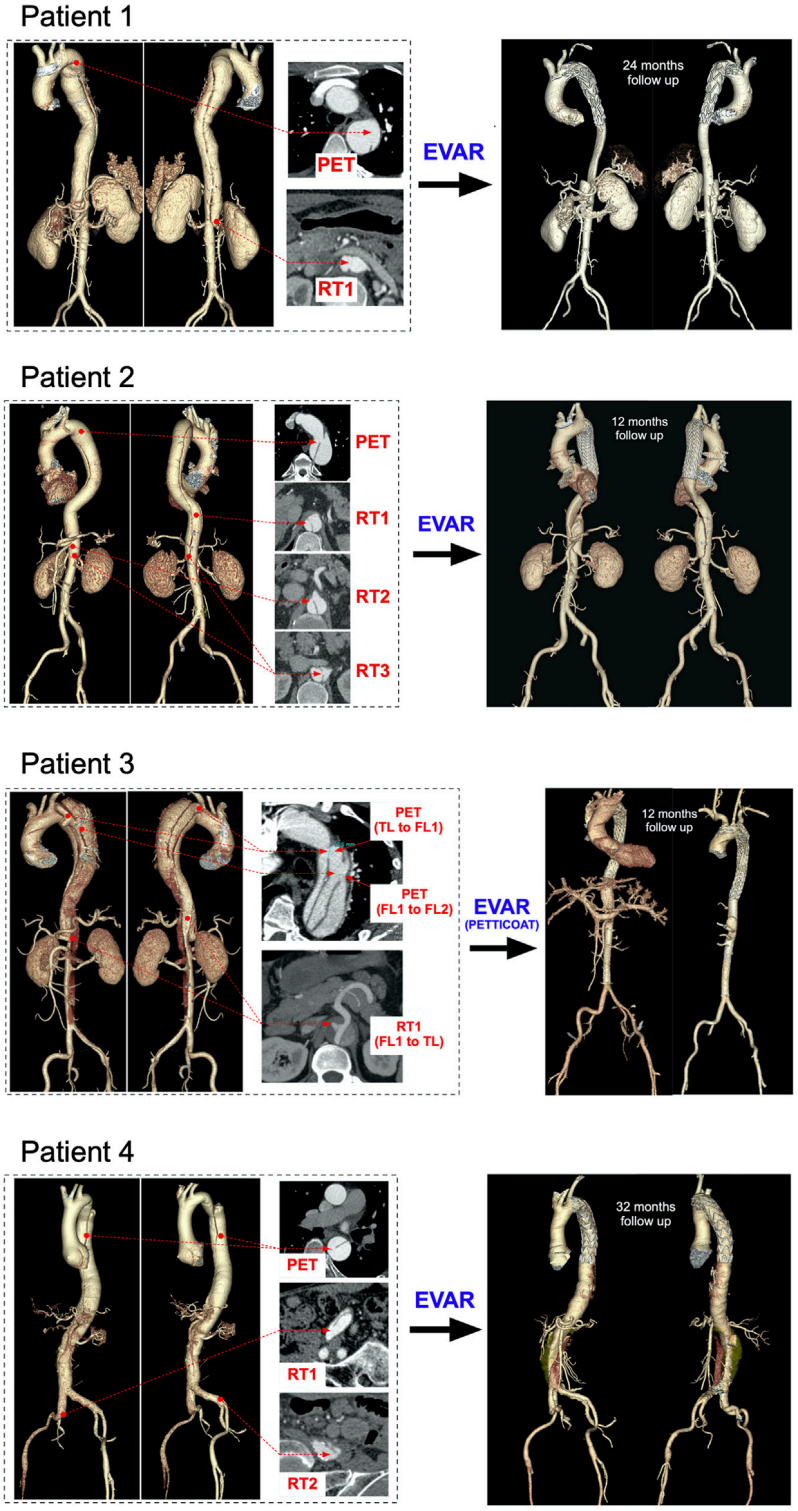
Aortic reconstructions **(Left)** pre-TEVAR volumetric aortic reconstructions for the four patients with selected cross-sections to point the visible primary entry tears (PET), re-entry tears (RT), true lumens (TL) and false lumens (FL) **(Right)** post-TEVAR volumetric aortic reconstructions for the four patients at 12-, 24- and 32 months follow-ups, respectively.

Patients underwent CT scans using a 320-row volumetric scanner (Aquilion ONE, Toshiba Medical System, Japan), generating axial images with 0.7 mm isotropic pixel spacing and 1 mm-thick sections from the top of the aortic arch to the bilateral iliac artery. Non-ionic contrast agent iopamidol 370 (iopamidol, Bracco, Milan, Italy) of 1.2 ml/kg followed by a saline flush of 25 ml was administered via antecubital vein using an automated injection system (Vistron CT Injection System, Medrad, Warrendale, PA, United States) at a flow rate of 4 ml/s. The trigger threshold was set at 200 HU (Aquilion ONE) within the descending aorta. For all patients, manual segmentation of the aortic geometries was performed by an expert operator using 3D Slicer (Kikinis et al., 2014). 3D renderings of the aortic anatomies are shown in Figure 1 together with tear locations and stent-graft positioning at follow-up. A set of geometric measurements associated with TBAD were taken from the CT images; these included PET area, re-entry tear (RT) maximum diameter, maximum FL cross-sectional area and TL and FL volumes. Perfusion of celiac artery, superior mesenteric artery, right and left renal arteries was assessed.

Geometric measurements taken from CT scans are reported in Table 1. For Patient 4, the FL covered a large portion of the abdominal aorta, even below the iliac bifurcation; therefore, TL and FL volumes were calculated from the PET to the celiac artery (CA) root.

### 4D Flow MRI Acquisition and Post-processing

**FIGURE 2:**
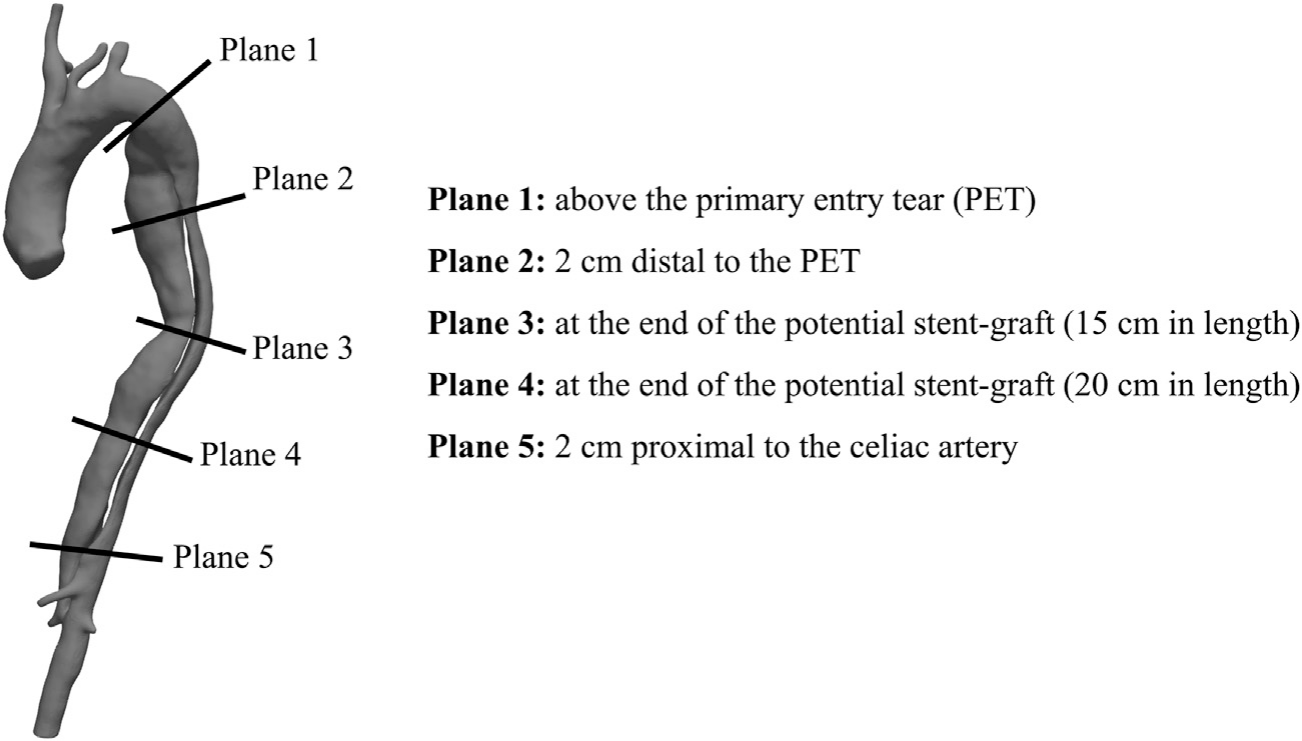
Cross-sections exemplification for post-processing **(Right)** Exemplification of the five cross-sectional planes selected along the aorta for 4D flow MRI analysis **(Left)** Volumetric reconstruction of a post-TEVAR aortic geometry (Patient 1) with stent-graft end pointed in red.

4D flow MRI images were acquired using a 3-T clinical MR scanner (Magnetom Avanto; Siemens Medical Solutions, Erlangen, Germany) using the following parameters: flip angle 7°; velocity encoding 150 cm/s in all directions; spatial resolution 1.85 × 1.85 × 2.5 mm^3^; and temporal resolution 47–61 ms. Retrospective electrocardiographic gating was used to reconstruct 12–20 frames/cardiac cycle. Using custom-built MATLAB code, the volumes segmented from CT scans were registered onto the 4D flow image volumes (Saitta et al., 2019). Data were visualized and further post-processed with Paraview (Kitware, NY, United States). Five cross-sectional planes were selected along the aorta for hemodynamics quantification (Figure 2).

### Endovascular Procedures

TEVAR was performed on all patients under general anesthesia using Valiant Capitivia (Medtronic, Minneapolis, MN) or C-TAG (W. L. Gore & Associates, Flagstaff, AZ) through the unilateral common femoral artery with a percutaneous puncture secured by two ProGlide stitches (Abbott Vascular, Abbott Park, IL). As previously reported (Guo et al., 2018), Patient 3, whose CT scan showed a TBAD with three-channel morphology, underwent TEVAR with a provisional extension to induce complete attachment (PETTICOAT) procedure (Melissano et al., 2012). Endograft oversizing was 0–5% according to the distance from the adventitia to the proximal landing zone. PETs were covered with a single 15- to 20 cm-long endograft. The proximal landing zone in Patient 1 was measured < 15 mm, the left subclavian artery was intentionally covered, as the right vertebral artery was patent and the left one was not dominant.

### Statistics

Hemodynamic and geometric measurements were entered into the statistical analysis software GraphPad Prism 8.1 (GraphPad Software, La Jolla, CA, United States of America). Variations in mean flow rate, peak flow rate, peak velocity magnitude and reverse flow index (RFI) in the TL and FL were compared using the paired *t*-test. A *p*-value of less than 0.05 was considered statistically significant.

## Results

**FIGURE 3:**
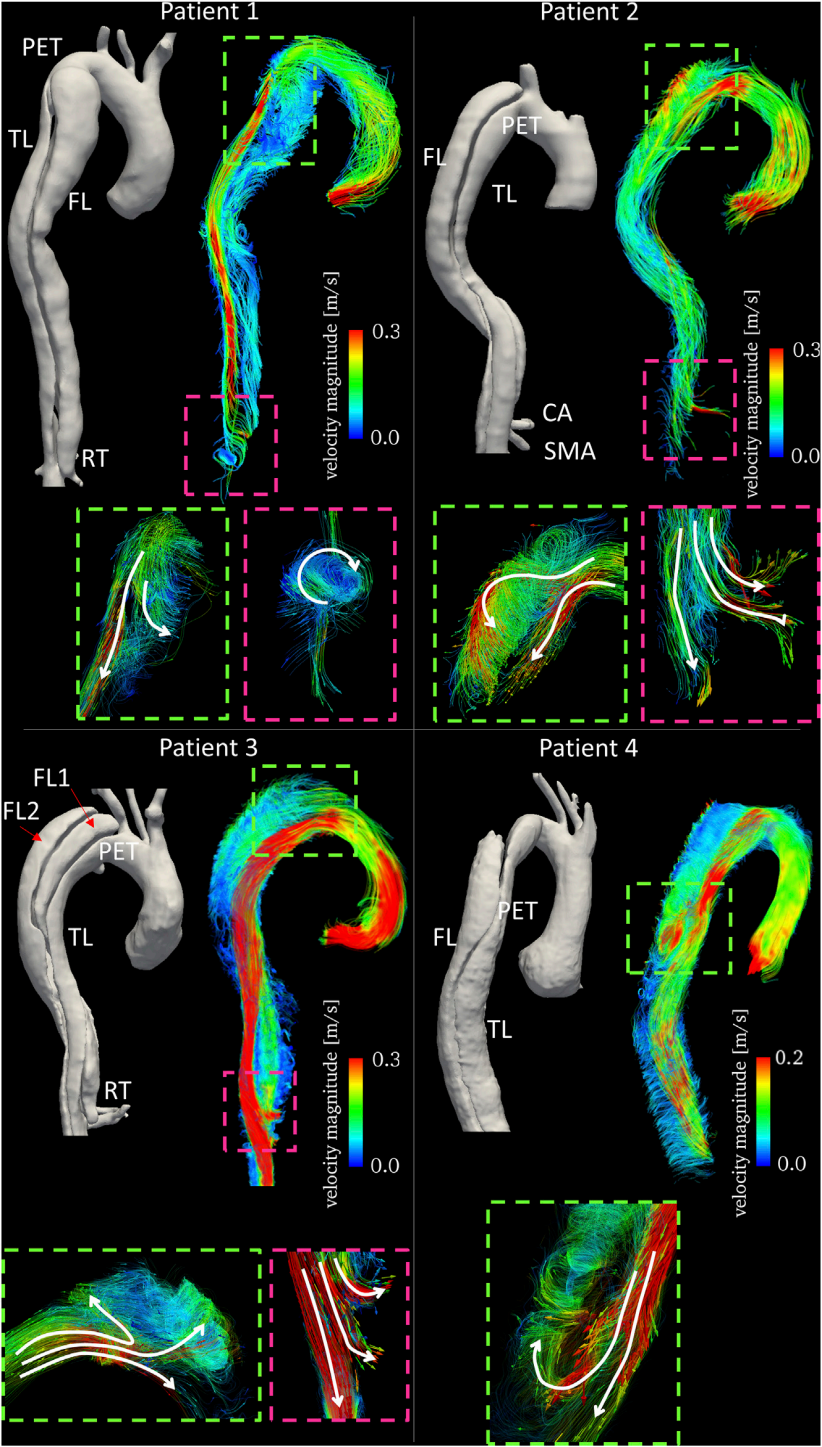
Time averaged velocity streamlines color-coded by velocity magnitude together with 3D aortic reconstructions for the four patients. Text labels indicating primary entry tears (PETs), true lumen (TL), false lumen (FL), celiac artery (CA) and superior mesenteric artery (SMA) are in white. Enlarged subfigures are shown at the primary entry tears (dashed green boxes) and re-entry tears (dashed pink boxes) with arrows to emphasize flow directionality.

Qualitative assessment of blood flow in the four patients was performed through visualization of streamlines within the aorta at mid-systole, emphasizing blood flow directionality around the PETs and RTs (Figure 3). Quantitative analysis of blood flow included the calculation of plane-averaged velocity magnitude, peak flow rate, peak velocity magnitude and RFI at five cross-sections along the aorta (Figure 2).

### 4D Flow-Based Qualitative Assessment

For all patients, visualization of streamlines revealed a clear difference in blood flow velocities within TL and FL, with TLs experiencing generally higher velocities. In Patient 1, flow patterns observed around the PET revealed the presence of a flow split, with a higher TL velocity due to compression, and lower velocity recirculating around the tear and entering the FL (Figure 3). Distal to the PET, no secondary tears were present, resulting in little flow disturbance in the TL. Blood flow acceleration was observed around the RET, with the formation of a high velocity stream re-entering the TL and causing helical flow formation.

In Patient 2, streamlines visualization showed a high velocity jet across the PET. This caused rapid deceleration of the flow jet in the proximal FL through the formation of vortical flow. Distal to the PET, the TL experienced significantly higher velocities due to the curvature of the aorta. In this case, both the CA and the superior mesenteric artery (SMA) were perfused by the FL. High velocity flow was observed entering the SMA from the FL through a re-entry tear (Figure 3).

Patient 3 featured a stark difference between FLs and TL in blood flow velocity along the aorta (Figure 3). In this case, the high velocity flow crossing the PET split three-ways, entering both FL1 and FL2 through the connected intimal tears. The high velocity flow leaving the TL and perfusing the SMA is shown in Figure 3; at this location, a re-entry tear (from FL1 to TL) was present.

Patient 4 presented a dissection with the PET located distal to the aortic arch. The high velocity flow in the TL at the arch and proximal to the PET entered the FL hitting the outer wall. A portion of the flow filled the upper region of the FL and the other portion flowed downstream. Stagnant flow was visible in the upper FL region (light blue streamline region in Figure 3).

### 4D Flow MRI-Based Quantitative Assessment

**FIGURE 4:**
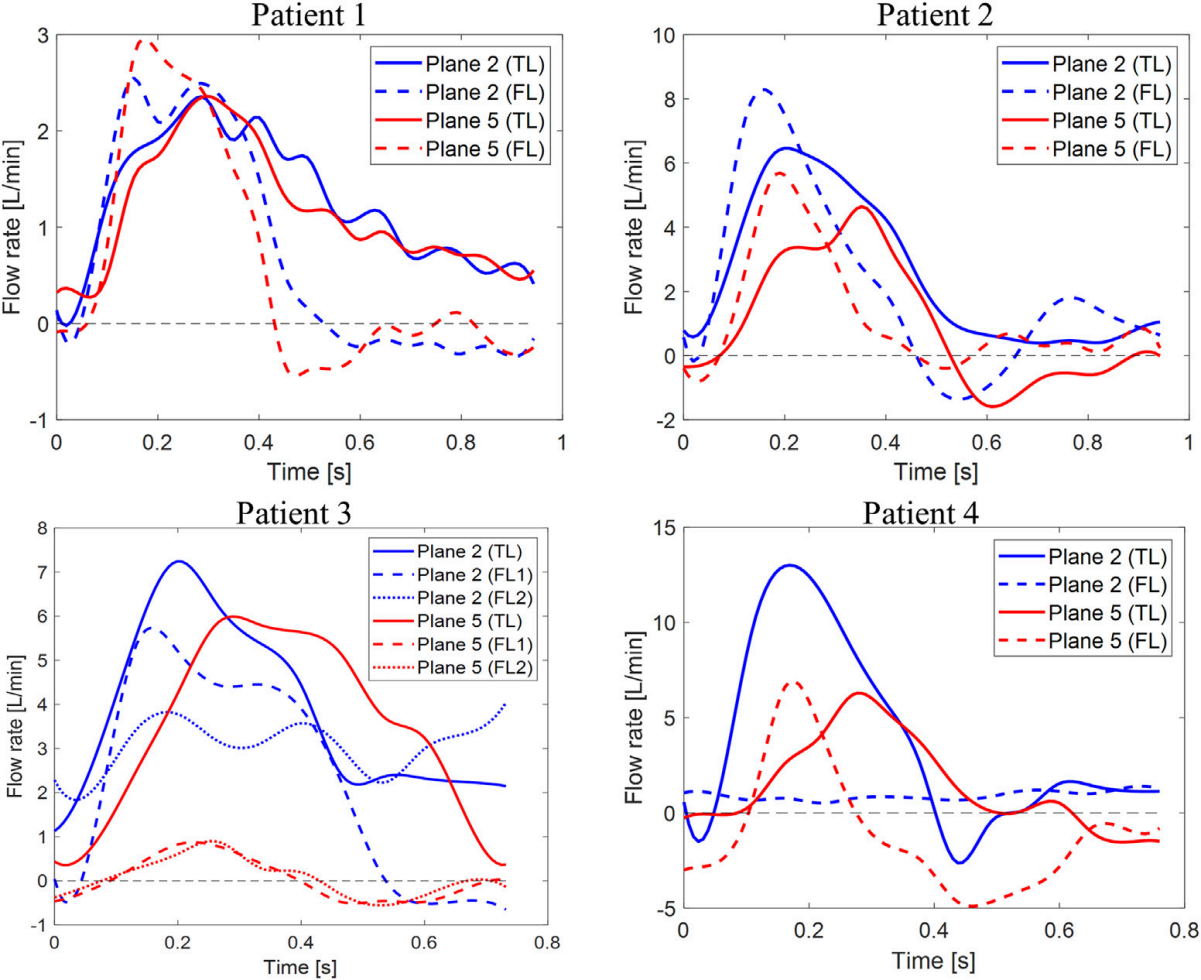
Flow rate waveforms. Time plots of flow rates calculated through planes two and five in the TL and FL for the four patients. *Y*-axis limits were adjusted to better show each patient’s waveforms.

Hemodynamic quantities of interest calculated on the five selected planes are reported in Table 2. For each plane, flow rate was calculated by taking the dot product between velocity and normal vectors and integrating the result over the plane area. In this way, a flow rate waveform was obtained for the cardiac cycle, from which peak and average values were computed. RFI was calculated as the fraction of negative area of the flow rate curve over the whole area under the curve (Birjiniuk et al., 2020).

$$RFI=\frac{\left|\int_{0}^{T}Q_{r}dt\right|}{\left|\int_{0}^{T}Q_{a}dt\right|+\left|\int_{0}^{T}Q_{r}dt\right|}\times 100\%,$$ (1)

where $Q_{r}$ and $Q_{a}$ are the total retrograde and antegrade flow rates, and T is the cardiac cycle period. Flowrate waveforms over time for planes 2 and 5 are plotted in Figure 4 for both TLs and FLs of the four patients.

**TABLE 2:** Blood flow measurements computed from 4D flow on the selected cross-sectional planes for the four patients.

Variable			Patient 1	Patient 2	Patient 3	Patient 4
Average velocity magnitude (cm/s)	plane 1	TL	13.2	13.6	22.7	10.0
	plane 2	TL	12.3	19.8	23.5	13.1
		FL	8.45	9.76	11.8 (FL1), 5.35 (FL2)	2.98
	plane 3	TL	10.5	12.3	37.0	9.00
		FL	4.95	8.37	14.0 (FL1), 3.76 (FL2)	8.76
	plane 4	TL	12.8	12.4	39.6	15.1
		FL	6.81	10.1	4.97 (FL1), 6.66 (FL2)	5.10
	plane 5	TL	13.5	14.3	124	5.06
		FL	7.20	6.08	8.48 (FL1), 10.8 (FL2)	1.22
Peak flow rate (L/min)	plane 1	TL	7.54	15.3	11.0	11.5
	plane 2	TL	2.35	6.46	7.25	13.0
		FL	2.55	8.29	5.73 (FL1), 4.04 (FL2)	1.40
	plane 3	TL	2.92	4.01	6.78	6.15
		FL	4.69	9.90	2.32 (FL1), 3.29 (FL2)	5.80
	plane 4	TL	2.52	4.58	5.19	6.17
		FL	4.24	10.0	2.87 (FL1), 2.79 (FL2)	7.03
	plane 5	TL	2.36	4.64	5.99	6.29
		FL	2.96	5.69	0.87 (FL1), 0.90 (FL2)	6.92
Peak velocity magnitude (cm/s)	plane 1	TL	30.8	39.7	40.6	29.9
	plane 2	TL	21.7	42.4	44.3	33.5
		FL	15.5	29.6	24.9 (FL1), 7.07 (FL2)	4.13
	plane 3	TL	21.6	38.8	70.5	23.3
		FL	15.9	27.4	24.6 (FL1), 6.72 (FL2)	22.4
	plane 4	TL	24.7	41.4	82.8	45.2
		FL	21.1	34.5	11.3 (FL1), 12.9 (FL2)	14.3
	plane 5	TL	27.1	37.8	208	160
		FL	19.6	20.7	15.8 (FL1), 23.0 (FL2)	67.8
Reverse flow fraction (%)	plane 1	TL	0	0	0	6.31
	plane 2	TL	0	0	0	6.17
		FL	11.7	7.51	5.51 (FL1), 0 (FL2)	0
	plane 3	TL	0	5.73	0	17.8
		FL	22.3	4.54	4.39 (FL1), 51.3 (FL2)	51.5
	plane 4	TL	0	10.0	0	7.64
		FL	9.37	1.87	3.35 (FL1), 59.4 (FL2)	61.4
	plane 5	TL	0	20.3	0	12.6
		FL	15.0	5.70	46.0 (FL1), 39.6 (FL2)	69.4

FL, false lumen; TL, true lumen.

On plane 1, proximal to the PET, the measured average velocity magnitudes varied between 10 and 22.7 cm/s. Null RFIs were found on plane 1 for Patients 1, 2 and 3, while a RFI = 6.3% was found for Patient 4. Such detail can also be appreciated in Figure 4, where null RFIs correspond to TL flow rate curves staying above the horizontal axis for the whole cardiac cycle.

For Patient 1, differences between TL and FL average velocity magnitudes increased with the distance from the PET and were equal to 3.85, 5.55, 5.99 and 6.30 cm/s for planes 2, 3, 4 and 5 respectively. Through all selected planes, peak flow rate values were larger in the FL than in the TL, with differences ranging from 0.19 to 1.76 L/min. TL peak velocity magnitudes were greater than FL values by 6.21, 5.69, 3.53 and 7.43 cm/s for planes 2, 3, 4 and 5, respectively.

For Patient 2, TL average velocity magnitudes were higher than FL values by 10.0, 3.93, 2.30 and 8.22 cm/s for planes 2 to 5. Peak flow rates were consistently higher in the FL, with differences ranging from −5.89 to −1 L/min. TL peak velocity magnitudes exceeded FL values by 12.8, 11.4, 6.84, and 17.2 cm/s for planes 2 to 5.

Patient 3 exhibited larger differences in average velocities between the TL and FL, with values ranging from 14.9 (on plane 2) to 114 cm/s (on plane 5). Peak flow rates were higher in the FL on planes 2 and 4 by 2.53 and 0.46 L/min respectively, whereas they were higher in the TL on planes 3 and 5 by 1.12 and 4.21 L/min. On all selected planes, peak velocity magnitudes were higher in the TL, with the largest difference of 185 cm/s found on plane 5. In this special case of TBAD, FL1 and FL2 average velocity magnitudes were averaged, while maximum values were considered for considering peak flow rates and peak velocity magnitudes.

For Patient 4, average velocity magnitude was higher in the TL for all planes, with values ranging from 5.06 (on plane 5) to 15.1 cm/s (on plane 4). On planes 2 and 3, TL peak flow rates were higher than FL values by 11.6 and 0.35 L/min, while on planes 4 and 5, FL values where greater than TL ones by 0.85 and 0.67 L/min, respectively. On all planes, peak velocity magnitudes were higher in the TL than in the FL, with differences ranging from 0.840 (on plane 3) to 92.2 cm/s (on plane 4).

Note that, given a VENC of 150 cm/s, fluid regions characterized by lower velocities are affected by noise in the velocity measurement around 7.50 cm/s (at 5% VENC).

### False Lumens Retrograde Flow Analysis

Differences between TL and FL can be visualized in Figure 4, where flow rates over time through planes 2 and 5 are plotted for the four patients. Negative flow rate curve areas correspond to non-zero RFI values. Patient 1 had null retrograde flow in the TL throughout the whole cardiac cycle. Retrograde flow in the FL during diastole resulted in 11.7 and 15% RFIs through planes 2 and 5. For Patient 2, a 7.5% RFI through plane 2 in the FL corresponded to retrograde flow during diastole, while a RFI of zero was found through plane 2 in the TL. An opposite trend was observed through plane 5, where retrograde diastolic flow was larger in the TL. TL and FL RFIs were 20 and 5.7%, respectively. For Patient 3, no retrograde flow was found in the TL across all planes. Similar flow rate waveforms were found for the two FLs at plane 5, where a peak value of 0.90 L/min was observed in FL1 and RFIs of 46 and 40% were found for FL1 and FL2, respectively. Higher FL flow rates were found across plane 2 than plane 5 throughout the cardiac cycle. Such a difference could be attributed to the fact that plane 2 is close to the PET where the flow is affected by the higher velocity in the TL.

Flow rate through Patient 4’s plane 2 in the TL displayed the typical aortic flow waveform, with clearly distinguishable systolic and diastolic phases. At plane 2, 80% of the total mean flow rate was carried by the TL and RFI = 0 was found in the FL. With respect to plane 2, the TL flow rate peak was observed after 0.11 s on plane 5, where retrograde diastolic flow corresponded to RFIs of 12.6 and 69.4% for the TL and FL, respectively.

Analysis of retrograde flow fraction revealed that patients 2 and 4 who had visceral branches perfused by the FL had non-zero RFIs in the TL.

### Statistical Analysis: Difference Between False Lumens and True Lumen

**FIGURE 5:**
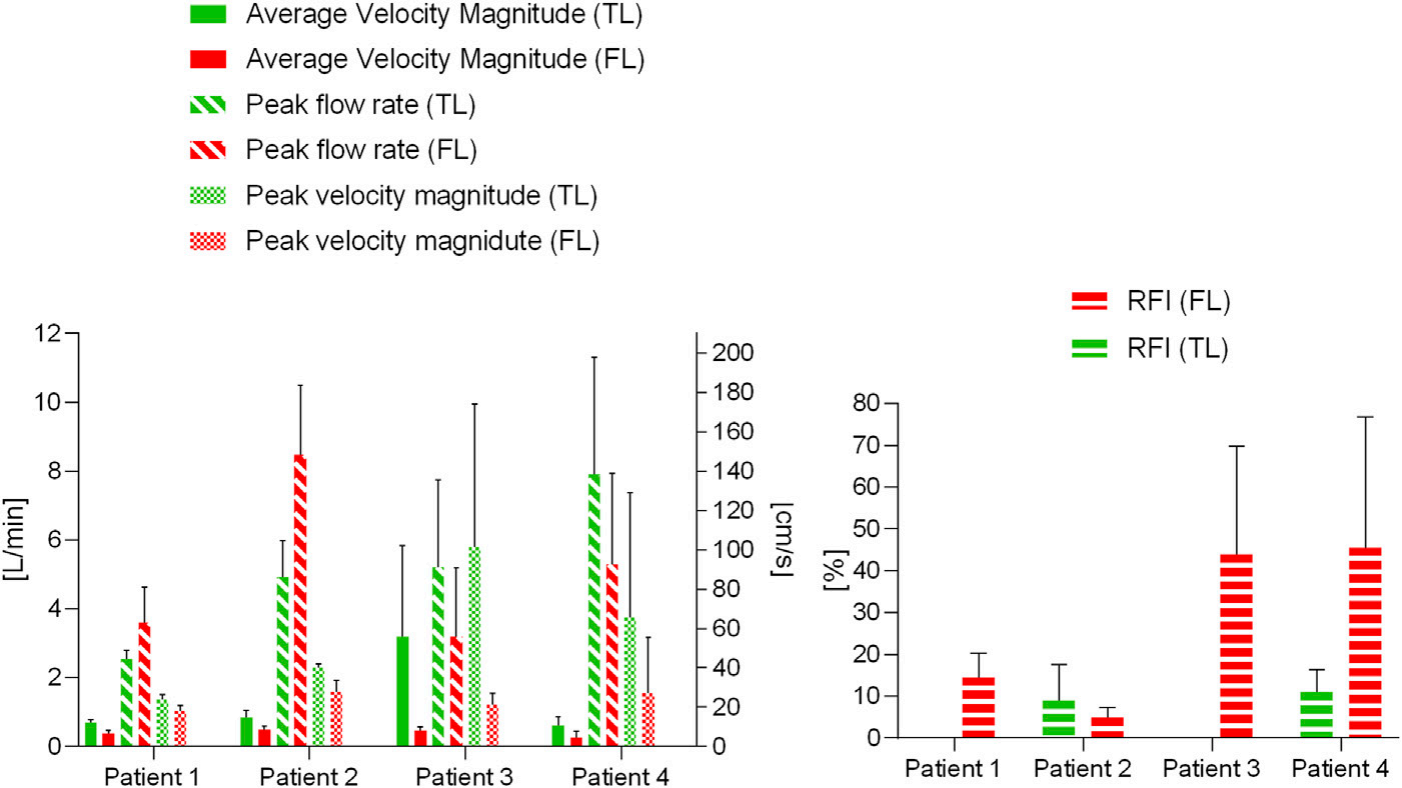
Blood flow parameter variations for the four patients in the TL and FL. Average velocity magnitude, peak flow rate and peak velocity magnitude are plotted on the left, reverse flow index (RFI) variations are plotted on the right.

The results of the statistical analysis to evaluate the differences in hemodynamics between TL and FL are reported in Table 3; Figure 5. Average velocity magnitude was significantly higher in the TL for Patient 1 (*p* < 0.005), Patient 2 (*p* < 0.05) and Patient 4 (*p* < 0.05), and higher, but not statistically significant, for Patient 3 (*p* = 0.08). For Patient 2, peak flow rate was larger in the FL (*p* < 0.05), whereas for Patient 3 it was found to be greater in the TL (*p* < 0.05). For all patients, peak velocity magnitude was higher in the TL, with statistically significant difference in Patient 1 (*p* < 0.05) and Patient 2 (*p* < 0.05). Patient 2 had higher TL RFIs, while all other patients exhibited greater RFIs in the FL (*p* < 0.005 for Patient 1, *p* < 0.05 for Patient 3 and *p* = 0.072 for Patient 4).

**TABLE 3:** Comparison of 4D flow analysis in the TL and FL among the four patients.

Variable		Patient 1	Patient 2	Patient 3	Patient 4
**Average velocity magnitude [cm/s]**	TL	12.3 ± 1.28	14.7 ± 3.52	56.03 ± 45.9	10.6 ± 4.46
	FL	6.85 ± 1.45	8.58 ± 1.83	8.23 ± 1.67^c^	4.52 ± 3.24
	T	5.61	3.09	2.08	2.81
	*P*	0.001^a^	0.02^a^	0.08	0.03^a^
**Peak flow rate [L/min]**	TL	2.54 ± 0.27	4.92 ± 1.06	6.30 ± 0.9	7.91 ± 3.40
	FL	3.61 ± 1.02	8.47 ± 2.02	3.20 ± 1.99^b^	5.29 ± 2.65
	T	2.03	3.12	2.85	1.22
	*P*	0.088	0.021^a^	0.029^a^	0.27
**Peak velocity magnitude [cm/s]**	TL	23.75 ± 2.62	40.10 ± 2.11	101.4 ± 73	65.5 ± 64
	FL	18.04 ± 2.79	28.05 ± 5.74	21.35 ± 5.69^b^	27.2 ± 28.1
	T	2.99	3.94	2.19	1.10
	*P*	0.024^a^	0.007^a^	0.07	0.31
**RFI [%]**	TL	0.004 ± 0.009	9.0 ± 8.56	0	11.0 ± 5.26
	FL	14.6 ± 5.65	4.9 ± 2.36	43.9 ± 0.26^c^	45.6 ± 31.3
	T	5.17	0.92	3.39	2.18
	*P*	0.002^a^	0.391	0.0146^a^	0.072

FL, false lumen; RFI, reverse flow index; TL, true lumen.

a Represents a statistically significant difference (*p* < 0.05). For Patient 3, a single asterisk

b Indicates that the mean of values calculated in the two FLs is reported, while a double asterisk

c Indicates that the maximum value between the two FLs is reported.

### Aortic Remodeling at Follow-Up

The four patients underwent midterm follow-up (12–32 months) CT scans (Figure 1). CT imaging demonstrated the good position of all endografts, obliteration of all FLs in the thoracic aorta, nearly complete thrombosis of the FLs, expansion of the TL, and no endoleak. In Patient 1 and Patient 2, the FL was almost thrombosed apart from the area near the distal re-entry tear. The right renal artery in Patient 1 was perfused by the FL with the flow from TL through the re-retry tear (RT1). In Patient 2, the re-entry tears (RT2 and RT3) played important roles in the blood flow exchange between TL and FL after TEVAR. This patient did not suffer malperfusion syndrome during follow up despite the CA and SMA were both perfused by FL. The FL collapsed and completely thrombosed in Patient 3 after the TEVAR with PETTICOAT procedure. However, a dynamic ischemia in the left renal artery became a static ischemia. Patient 4 had a better TL remodeling in thoracic aorta than the abdominal aorta. The abdominal FL was perfused through the two distal re-entry tears in the iliac arteries and partial thrombosed. Unfortunately, the left renal artery was malperfused during follow up. The post-TEVAR analysis is listed in Table 4.

**TABLE 4:** Geometric analysis for the four kind of aortic dissection post-TEVAR.

Variable	Patient 1	Patient 2	Patient 3	Patient 4
Follow up	24 months	12 months	12 months	32 months
PET maximum diameter	Covered	Covered	Covered	Covered
RT maximum diameter (mm)	8.98 (RT1)	8.51 (RT1)	Covered	4.44 (RT1)
		4.37 (RT2)		7.14 (RT2)
		5.27 (RT3)		
Celiac artery	Perfused	Perfused by FL	Perfused	Perfused
Superior mesenteric artery	Perfused	Perfused by RT2	Perfused	Perfused
Right renal artery	Perfused through RT1	Perfused	Perfused	Perfused
Left renal artery	Perfused	Perfused through RT3	Malperfused static ischemia	Malperfused
FL area	Decreased	Decreased	Collapsed	Decreased
TL volume	Increased	Increased	Increased with remodeling	Increased
FL volume	Almost thrombosed apart from the area near the RT1	Partially thrombosed	Collapsed	Completely thrombosed in TA, and partially thrombosed in AA

FL, false lumen; PET, primary entry tear; RT, re-entry tear; TL, true lumen; TA, thoracic aorta; AA, abdominal aorta.

## Discussion

Current guidelines suggest that non-invasive methods based on CFD and MRI can provide additional dynamic information regarding entry tear flow or arterial vessel involvement (François et al., 2013; Riambau et al., 2017). However, it is still unclear how blood flow distributes at the entry/re-entry tear sites in TBAD. In the present study, four cases of TBAD with different morphologies were analyzed using 4D flow MRI for qualitative and quantitative blood flow assessment. Additionally, observations related to aortic remodeling for the four patients post-TEVAR were reported. This analysis demonstrates the added value of using 4D flow MRI to provide clinically relevant information for better understanding of blood flow alterations in the TL and FL, which can vary among different types of TBADs.

Patient-specific analyses using flow visualization and quantification would aid physicians understand the complex pathologic changes of TBAD with different morphologies. However, interpreting the meaning of different hemodynamic measures is particularly challenging, and most studies report analyses for only one or a few patients with no follow-up for validation (Müller-Eschner et al., 2011).

This study confirms the feasibility of using 4D flow MRI to assess hemodynamic differences between TL and FL. The presence of blood flow in the FL has been shown to be the most important risk factor for FL enlargement (Sueyoshi et al., 2004). In particular, Inoue et al. (Inoue et al., 2000) found volume flow rate in the FL to be a significant predictor of FL growth; the larger the flow rate, the faster was the FL expansion. In the present study, 4D flow-derived velocity streamlines showed the formation of vortical flow structures in the FL near the PETs caused by an acceleration of blood flow entering the FL. These observations are in agreement with findings by François et al. (2013) and Müller-Eschner et al. (2011), who reported the presence of abnormal, complex flow patterns in the FL. In general, blood flow in the FL was characterized by lower velocities and higher RFIs. In our study, average velocity magnitude was found to be significantly higher (*p* < 0.05) in the TL for three of the four patients analyzed (*t* = 5.61, 3.09 and 2.81 m/s for Patients 1, 2 and 4 respectively). Similar results were found by Liu et al. (2018), who analyzed a cohort of 16 patients and reported higher average velocities in the TL (t = 7.64) and higher RFIs in the FL (*t* = 5.01).

A second key finding of this study was based on the retrograde flow analysis performed on the four patients. RFIs were significantly higher in the FL (*p* < 0.05) for Patient 1 (*t* = 5.17) and Patient 3 (*t* = 3.39). Interestingly, a correlation between RFIs and pre-TEVAR side branches perfusion was found. No reversed flow was observed in the TL of Patients 1 and 3, who concurrently had the CA perfused by the TL. On the other hand, Patients 2 and 4 exhibited non-zero TL RFIs and had the CA and left renal artery perfused by the FL. These findings support the previously postulated hypothesis that reversed flow may be related to distal reentry pathways into the FL with higher pressure, causing disturbances of blood stream and retrograde flow in the TL (Catapano et al., 2020). This correlation is further backed up by the overall higher RFI in the TL than in the FL of Patient 2 (Figure 5). In this case in fact, perfusion of CA, SMA and left renal artery was provided by the FL.

An additional aim of this study was to explore whether specific hemodynamic conditions pre-TEVAR could potentially be related to aortic remodeling post-TEVAR. It seems likely that incorporating hemodynamic parameters obtained with 4D flow MRI will improve predictions. A recent 4D flow MRI-based patient-specific study by Takei et al. (2019) revealed that flow rate at the re-entry site and in the FL can have important effects on FL volume change. To better manage distal re-entry tears and avoid malperfusion, fenestrated and branched stent grafts have been used to treat patients (Oikonomou et al., 2014; Pellenc et al., 2019). In two of the four cases examined here, aortic complications such as partial thrombosis of FL and organ malperfusion were observed during follow-up. The limited number of patients in our study did not allow for a predictive analysis. However, they did provide useful insights about hemodynamics and potential associations with aortic remodeling during follow-up. Patients 1 and 2, had similar TL and FL flow rate waveforms (and peaks) during systole, but differed during diastole (Figure 3). In both patients, peak flow rates were higher in the FL than in the TL. On the other hand, the remarkable difference in pre-intervention flow rates observed in Patient 3 (especially on Plane 5), could be linked to the static ischemia developed in this patient. In this case, pre-TEVAR TL blood flow was characterized by significantly higher average velocities and flow rates and lower RFIs. The low FL flow rate and the intricate double FL morphology led to the complete collapse of the FL after TEVAR with distal bare stents placement preventing perfusion of the left renal artery and eventually leading to ischemia at follow-up. Similarly, Patient 4 was characterized by a lower peak flow rate in the FL with respect to the TL (Figure 5). In this case, the left renal artery was perfused by the FL pre-TEVAR and suffered from malperfusion after intervention. Our findings suggest that if peak flow rate before TEVAR is lower in the FL than in the TL, the FL may collapse or thrombose after occlusion of the PET by the graft; if this condition occurs concurrently with branch vessel perfusion by FL through a RT, malperfusion might be observed at follow-up.

Aortic hemodynamics is the key to aortic remodeling in both acute and chronic TBAD, as well as aortic-related complications after TEVAR (Tsai et al., 2007). Therefore, identifying and validating imaging biomarkers of aortic remodeling would have significant clinical implications. In our current study, it is difficult to draw robust conclusions on which specific hemodynamic parameters are associated with the clinical outcomes observed at follow-up. Future studies will include a larger patient cohort to further investigate potential predictive blood flow features using 4D flow MRI or CFD, with the goal of helping clinicians in treatment planning for TBAD patients.

Despite providing key information about patient-specific hemodynamics, 4D flow MRI presents some inherent limitations. The relatively high *VENC* of 150 cm/s, necessary to avoid aliasing in the TL, leads to lower signal-to-noise ratio in the FL which is characterized by lower velocities (François et al., 2013). The use of multi-*VENC* 4D flow MRI, could potentially solve this problem, and it seems especially necessary to evaluate blood flow in TBADs (Nett et al., 2012; Ha et al., 2016). Additionally, 4D flow MRI techniques are affected by errors due to noise and limited spatial and temporal resolution (Markl et al., 2016). Limitations in spatial resolution have been shown to have a significant effect on near-wall flow parameters such as wall shear stress (Piatti et al., 2017). Nonetheless, directly measured parameters such as flow rate through a plane are less sensitive to this constraint and can be estimated with reasonable accuracy (David et al., 2019). On the other hand, a low temporal resolution may lead to underestimations of the true peak velocity and flow rate. In this regard, new phase-contrast MR sequences hold the potential to increase temporal resolution while keeping scan time to a minimum. Finally, the accuracy of geometric measurements taken on CT images inevitably depends on the image voxel size (Horner et al., 2019). Nonetheless, since no quantitative correlations were made between geometric features and flow parameters, our analyses are not significantly affected by intrinsic errors due to voxel size.

This study demonstrated the clinical applicability of 4D flow MRI in the context of TBAD, paving the way for future studies involving larger patient cohorts. Through 4D flow analysis, it was possible to quantify hemodynamics of TL vs. FL, showing the importance of retrograde flow in relation to visceral branch vessel perfusion. Our results confirm the potential of 4D flow MR imaging to be used as a non-invasive technique for patient risk stratification and treatment planning.

## Data Availability

The data that support the findings of this study are available from the corresponding author, BG, upon reasonable request.
